# European LeukemiaNet 2020 recommendations for treating chronic myeloid leukemia

**DOI:** 10.1038/s41375-020-0776-2

**Published:** 2020-03-03

**Authors:** A. Hochhaus, M. Baccarani, R. T. Silver, C. Schiffer, J. F. Apperley, F. Cervantes, R. E. Clark, J. E. Cortes, M. W. Deininger, F. Guilhot, H. Hjorth-Hansen, T. P. Hughes, J. J. W. M. Janssen, H. M. Kantarjian, D. W. Kim, R. A. Larson, J. H. Lipton, F. X. Mahon, J. Mayer, F. Nicolini, D. Niederwieser, F. Pane, J. P. Radich, D. Rea, J. Richter, G. Rosti, P. Rousselot, G. Saglio, S. Saußele, S. Soverini, J. L. Steegmann, A. Turkina, A. Zaritskey, R. Hehlmann

**Affiliations:** 1 Klinik für Innere Medizin II, Universitätsklinikum, Jena, Germany; 2 Department of Hematology/Oncology, Policlinico S. Orsola-Malpighi, University of Bologna, Bologna, Italy; 3 Weill Cornell Medical College, New York, NY USA; 4 Karmanos Cancer Center, Detroit, MI USA; 5 Hammersmith Hospital, Imperial College, London, UK; 6 Hospital Clinic IDIBAPS, Barcelona, Spain; 7 Department of Molecular & Clinical Cancer Medicine, University of Liverpool, Liverpool, UK; 8 Georgia Cancer Center, Augusta University, Augusta, GA USA; 9 Huntsman Cancer Center Salt Lake City, Salt Lake City, UT USA; 10 Centre Hospitalier Universitaire de Poitiers, Poitiers, France; 11 Norwegian University of Science and Technology, Trondheim, Norway; 12 South Australian Health and Medical Research Institute, Adelaide, SA Australia; 13 Amsterdam University Medical Center, VUMC, Amsterdam, The Netherlands; 14 MD Anderson Cancer Center, Houston, TX USA; 15 St. Mary´s Hematology Hospital, The Catholic University, Seoul, Korea; 16 University of Chicago, Chicago, IL USA; 17 University of Toronto, Toronto, Canada; 18 Institut Bergonie, Université de Bordeaux, Bordeaux, France; 19 Department of Internal Medicine, Masaryk University Hospital, Brno, Czech Republic; 20 Centre Léon Bérard, Lyon, France; 21 Universitätsklinikum, Leipzig, Germany; 22 Department Clinical Medicine and Surgery, University Federico Secondo, Naples, Italy; 23 Fred Hutchinson Cancer Center, Seattle, WA USA; 24 Hôpital St. Louis, Paris, France; 25 University of Lund, Lund, Sweden; 26 Centre Hospitalier de Versailles, University of Versailles Saint-Quentin-en-Yvelines, Versailles, France; 27 University of Turin, Turin, Italy; 28 III. Medizinische Klinik, Medizinische Fakultät Mannheim, Universität Heidelberg, Mannheim, Germany; 29 Hospital de la Princesa, Madrid, Spain; 30 National Research Center for Hematology, Moscow, Russian Federation; 31 Almazov National Research Centre, St. Petersburg, Russian Federation; 32 ELN Foundation, Weinheim, Germany

**Keywords:** Chronic myeloid leukaemia, Targeted therapies

## Abstract

The therapeutic landscape of chronic myeloid leukemia (CML) has profoundly changed over the past 7 years. Most patients with chronic phase (CP) now have a normal life expectancy. Another goal is achieving a stable deep molecular response (DMR) and discontinuing medication for treatment-free remission (TFR). The European LeukemiaNet convened an expert panel to critically evaluate and update the evidence to achieve these goals since its previous recommendations. First-line treatment is a tyrosine kinase inhibitor (TKI; imatinib brand or generic, dasatinib, nilotinib, and bosutinib are available first-line). Generic imatinib is the cost-effective initial treatment in CP. Various contraindications and side-effects of all TKIs should be considered. Patient risk status at diagnosis should be assessed with the new EUTOS long-term survival (ELTS)-score. Monitoring of response should be done by quantitative polymerase chain reaction whenever possible. A change of treatment is recommended when intolerance cannot be ameliorated or when molecular milestones are not reached. Greater than 10% BCR-ABL1 at 3 months indicates treatment failure when confirmed. Allogeneic transplantation continues to be a therapeutic option particularly for advanced phase CML. TKI treatment should be withheld during pregnancy. Treatment discontinuation may be considered in patients with durable DMR with the goal of achieving TFR.

## Introduction

The life expectancy of a newly diagnosed patient with Philadelphia chromosome-positive (Ph+), BCR-ABL1+ chronic myeloid leukemia (CML) in chronic phase (CP) is now very close to that of age matched individuals in the general population, at least in western countries [1–5]. This remarkable achievement reflects the success of a large number of prospective clinical trials, summarized in management recommendations previously published by the European LeukemiaNet (ELN) [6–8]. Several tyrosine kinase inhibitors (TKIs) are approved for the treatment of CML and the choice of therapies for individual patients is determined by considerations of efficacy, tolerability, early and late toxicity and drug costs. More recently, there has been increasing focus on quality of life and avoiding long-term organ toxicities and, in particular, the identification of strategies to maximize the possibility of stopping TKI therapy resulting in so-called “treatment-free remission” (TFR). However, in resource-poor countries, the availability of effective drugs and essential monitoring may be limited, and the goal of treatment remains survival [9].

Since management goals and therapeutic scenarios continue to evolve, the ELN again appointed an international panel of experts to update the previous recommendations [8]. The current recommendations are based insofar as possible on high-quality evidence reported in peer-reviewed journals, supplemented by the consensus of the panel. The recommendations are geared towards those medical professionals treating patients with CML and towards concerned patients to provide better understanding of their disease and their treatment.

## Methods

The ELN panel for recommendations in CML comprises 34 experts from Europe, America, and the Asian-Pacific areas. The panel met six times at international meetings of the American Society of Hematology (2015, 2016), the European School of Hematology (2017), the ELN (2019), the European Investigators on CML (2019), and the European School of Hematology/International CML Foundation (2019). Five sets of key questions were submitted to panel members to complement the meetings. Discordant opinions were harmonized by email discussions, and a consensus of 75–100% was reached in most, but not all instances. Unresolved controversies are described in the discussion section. The costs of the meetings and the preparation of the interim and final reports were born entirely by ELN, a research network of excellence initiated by the European Union, now funded by donations and projects of ELN participants. There was no financial support from industry for any activity. Treatment recommendations are limited to the TKIs that have been approved with at least one indication in CML, either by the US Federal Drug Administration (FDA) or/and by the European Medicine Agency (EMA). The drugs are listed in the order of FDA approval. The panel acknowledges that not all drugs may be available worldwide and that the high price of some makes access to these drugs problematic in some countries.

Relevant papers published after the third version of the ELN recommendations in 2013 were identified through the PubMed database and were critically reviewed. With few exceptions, only papers published after 2013 are cited here. The panel also reviewed and utilized as appropriate abstracts presented at the recent meetings of the American Society of Clinical Oncology, the European Hematology Association and the American Society of Hematology.

The definitions of hematologic, cytogenetic, and molecular responses were maintained from previous versions [6–8].

## Clinical care

The goal of CML treatment is normal survival and good quality of life without life-long treatment [2, 4, 10, 11]. Specialty care for complications of chronic TKI therapy is required for optimal management. CML is no longer a disease that is fatal in a few years. Rather, it has evolved into a chronic condition with age-related comorbidities. Leukemia, therefore, is no longer the primary cause of disability and death. CML, a rare disease, requires specific competence to deal with its treatment and its complications [12]. The therapy is expensive and life-saving, but only if planned and supervised by trained clinicians. Treatment should be managed in cooperation with a specialized referral center where there is rapid access to quality-controlled, reliable tests including chromosome banding analysis (CBA), fluorescence in situ hybridization (FISH) for specific cases, and quantitative reverse transcriptase polymerase chain reaction (qPCR) with mutation analysis (Sanger or next generation sequencing, NGS) [12].

## Diagnostic work-up

When CML is diagnosed, a bone marrow aspirate is required for morphology, since the proportion of blast cells and of basophils is important to distinguish CP from accelerated phase (AP) and blastic phase (BP), and for cytogenetics. A core biopsy may be done to evaluate the degree of fibrosis which has prognostic importance and may identify nests of blasts not evident in the aspirate [13–15]. Cytogenetics should be performed by CBA of Giemsa-stained metaphases from bone marrow cells. A qualitative reverse transcriptase PCR on peripheral blood cells is mandatory to identify the type of BCR-ABL1 transcripts that can be appropriately followed when assessing response to TKI therapy. About 2–4% of patients harbor atypical BCR-ABL1 transcripts lacking ABL1 exon2 (e13a3 or e14a3) or resulting from atypical BCR breakpoints (e.g., e1a2, e6a2, e8a2, or e19a2) that may yield a false negative PCR using routine primer/probe sets in qualitative or quantitative reverse transcriptase PCR protocols. If not tested at diagnosis, a false impression may be given that a patient is in complete molecular response after TKI treatment. A quantitative PCR is not mandatory at diagnosis. If a molecular assay demonstrates BCR-ABL1, but the Ph-chromosome cannot be identified by cytogenetics, a FISH test is required. The diagnostic work-up is completed by a physical examination with particular reference to spleen and liver size, a standard biochemical profile including hepatitis B serology, cholesterol, lipase, and hemoglobin A1c values, and an electrocardiogram (Table 1).

**Table 1:** Diagnostic work-up, baseline.

Physical examination with particular reference to spleen and liver size
Complete blood cell count with microscopic differential
Bone marrow aspirate for cytologic examination and cytogenetics; core biopsy if dry tap
Chromosome banding analysis (CBA)
Fluorescence in-situ hybridization (FISH) only in case of Ph-negativity
Qualitative reverse transcriptase polymerase chain reaction (PCR) for the detection of BCR-ABL1 transcripts and identification of the transcript type
Electrocardiogram
Standard biochemical profile with hepatitis B-serology

Performing other tests and diagnostic procedures depends on characteristics of the individual patient, medical history, and comorbidities.

The definitions of CP, AP, and BP are based on hematologic and clinical parameters and unchanged from previous published versions [6–8]. However, resistance to two TKIs, detection of a BCR-ABL1 kinase domain (KD)-mutation, or emergence of additional chromosome abnormalities in Ph+ cells (ACA) raise concerns for progression of disease [16–19].

## Epidemiology

In western countries the median age of CML patients is about 57 years [4, 19, 20]. Patients older than 70 years make up more than 20% and children and adolescents <5%.

In Asia and in Africa the median age at diagnosis is <50 years, reflecting in part the lower median age of the population [9]. Age is an important variable in the choice of treatment, because overall survival (OS), comorbidities, and the development of complications are all age-related. TFR may be the main goal for any patient, irrespective of age, but it is clear that the younger the patient the stronger the case for achieving TFR. The recommendations presented here are primarily intended for the use in adult patients, as the biology of pediatric patients and their treatment exhibit unique characteristics.

## Baseline prognostic factors

Three prognostic systems, Sokal, Euro, and EUTOS, arithmetically derived, but based on simple clinical and hematologic data, have been used to estimate the survival risk at baseline [21–23]. The Sokal score has been particularly popular and was used in most TKI-trials [21]. These risk scores were designed to evaluate differences in survival [21, 22] or response [23]. Since most patients now die from causes other than leukemia while still in remission, a fourth risk score has been developed to predict the probability of dying from CML (leukemia-related death, LRD): the new EUTOS Long Term Survival (ELTS) score [24–26]. The ELTS score, based on TKI-treated patients, uses the same simple hematologic data, spleen size, and age as Sokal. The main difference is in the negative prognostic value of age, since it has less impact in TKI-treated patients (ELTS) than in patients treated with conventional chemotherapy (Sokal). The Sokal score apportions more patients to the intermediate- and high-risk groups than ELTS, particularly among older patients (Table 2).

**Table 2:** Prognostic scores at baseline and comparison of Sokal [21] and ELTS [24] scores.

(a) Score calculation		
Score	Calculation	Definition of risk groups
Sokal	Exp 0.0116 × (age—43.4)	Low risk: <0.8
	+0.0345 × (spleen—7.51)	Intermediate-risk: 0.8–1.2
	+0.188 × [(platelet count/700)^2^—0.563]	High-risk: >1.2
	+0.0887 × (blood blasts—2.10)	
ELTS	0.0025 × (age/10)^3^	Low risk: <1.5680
	+0.0615 × spleen size	Intermediate risk:1.5680–2.2185
	+0.1052 × peripheral blood blasts	High-risk: >2.2185
	+0.4104 × (platelet count/1000)^–0.5^	

(b) Risk strata proportions and outcome						
	Low risk		Intermediate risk		High risk	
*n* = 5154	Sokal	ELTS	Sokal	ELTS	Sokal	ELTS
%	38	55	38	28	23	13
10-year OS	89%	88%	81%	79%	75%	68%
6-year LRD	3%	2%	4%	5%	8%	12%

*ELTS* EUTOS score for long-term survival considering leukemia-related death (LRD); *OS* overall survival; age given in years; spleen size in cm below costal margin measured by palpation (maximum distance); blasts in percent of peripheral blood differential; platelet count, 10^9^/L. All values are pre-treatment. To calculate Sokal and ELTS scores, go to: http://www.leukemia-net.org/content/leukemias/cml/elts_score/index_eng.html.

The panel recommends the use of the new ELTS survival score. Prognostic scores at baseline of CP CML and calculation of relative risk as well as a comparison of Sokal and ELTS scores are shown in Table 2 [21, 24].

A score calculator is accessible via http://www.leukemia-net.org/content/leukemias/cml/elts_score/index_eng.html.

Several additional risk factors have been identified, but so far none has been validated and found useful with the exception of fiber content in bone marrow biopsies [13–15] and high-risk ACA [16–18]. These include +8, a second Ph-chromosome (+Ph), i(17q), +19, −7/7q-, 11q23, or 3q26.2 aberrations, and complex aberrant karyotypes. High-risk ACA predict a poorer response to TKIs and a higher risk of progression [16–18]. In the last version of the recommendations, ACA were mentioned as a “warning” [8]. Currently, the panel recommends classifying ACA and treating patients with high-risk ACA as high-risk patients.

## Molecular response definitions

Molecular response must be assessed according to the International Scale (IS) as the ratio of BCR-ABL1 transcripts to ABL1 transcripts, or to other internationally accepted control transcripts (e.g., beta glucuronidase, GUSB, for testing high BCR-ABL1 levels at baseline, after relapse, or in advanced disease), and must be expressed and reported as BCR-ABL1 % on a log scale, where 1%, 0.1%, 0.01%, 0.0032%, and 0.001% correspond to a decrease of 2, 3, 4, 4.5, and 5 logs, respectively, below the standardized baseline that was used in the IRIS study [27–29]. BCR-ABL1 ≤ 1% is equivalent to complete cytogenetic remission, CCyR [30]. BCR-ABL1 transcript level ≤0.1% is defined as major molecular response (MMR) or MR^3^. A BCR-ABL1 transcript level ≤0.01% or undetectable disease in cDNA with >10,000 ABL1 transcripts is defined as MR^4^. A BCR-ABL1 transcript level ≤0.0032% or by undetectable disease in cDNA with >32,000 ABL1 transcripts in the same volume of cDNA used to test for BCR-ABL1 is defined as MR^4.5^. Assay sensitivity should be defined in a standardized manner when BCR-ABL1 mRNA is undetectable. The term “complete molecular response” should be avoided and substituted by the term “molecularly undetectable leukemia” with specification of the number of the control-gene transcripts. These working definitions depend critically on the ability of testing laboratories to measure absolute numbers of control-gene transcripts in a comparable manner (Table 3) and on their ability to achieve the PCR sensitivity required for BCR-ABL1 detection [31, 32].

**Table 3:** Reference gene numbers required for scoring molecular response [29, 31].

	MMR	MR^4^	MR^4.5^	MR^5^
Minimum sum of reference gene transcripts	10,000 ABL1^a^ 24,000 GUSB^a^	10,000 ABL1 24,000 GUSB	32,000 ABL1 77,000 GUSB	100,000 ABL1 240,000 GUSB
BCR-ABL1 transcript level on the IS^b^	≤0.1%	≤0.01%	≤0.0032%	≤0.001%

^a^Minimal sensitivity for accurate quantification.

^b^International Scale, IS.

## Monitoring, response to treatment, and milestones

Blood cell counts and differential cell counts are required every 2 weeks until a complete hematologic response is achieved or, more frequently, in the event of hematologic toxicity. QPCR on blood cells, expressed as BCR-ABL1 % according to the IS, must be performed at least every 3 months even after an MMR is achieved and confirmed, because close monitoring of molecular response is required to assess eligibility for treatment discontinuation. Cytogenetics, by CBA of marrow cell metaphases, may be useful when performed, but alone is not sufficiently sensitive to monitor response. However, cytogenetics should be done in patients with atypical translocations, rare or atypical BCR-ABL1 transcripts that cannot be measured by qPCR, treatment failure/resistance to exclude ACA, and with progression to AP or BP. FISH monitoring may be needed in patients with atypical transcripts.

The monitoring milestones of BCR-ABL1 transcript levels by the IS at 3, 6, and 12 months (Table 4) determine whether the current treatment should be continued (optimal response), changed (failure/resistance), or carefully considered for continuation or change, depending on patients´ characteristics, comorbidities and tolerance (warning). Additional qPCR testing may be indicated if the kinetics of the response are not clear, or if toxicity or intolerance cause dose interruptions or reductions. The same definitions are recommended for second-line treatment. Achieving an MMR (BCR-ABL1 ≤ 0.1%) predicts a CML-specific survival close to 100% as disease progression is uncommon once this level of cytoreduction has been achieved.

**Table 4:** Milestones for treating CML expressed as BCR-ABL1 on the International Scale (IS).

	Optimal	Warning	Failure
Baseline	NA	High-risk ACA, high-risk ELTS score	NA
3 months	≤10%	>10%	>10% if confirmed within 1–3 months
6 months	≤1%	>1–10%	>10%
12 months	≤0.1%	>0.1–1%	>1%
Any time	≤0.1%	>0.1–1%, loss of ≤0.1% (MMR)^a^	>1%, resistance mutations, high-risk ACA

For patients aiming at TFR, the optimal response (at any time) is BCR-ABL1  ≤ 0.01% (MR^4^).

A change of treatment may be considered if MMR is not reached by 36–48 months.

*NA* not applicable, *ACA* additional chromosome abnormalities in Ph+ cells, *ELTS* EUTOS long term survival score.

^a^Loss of MMR (BCR-ABL1 > 0.1%) indicates failure after TFR

## First-line treatment

With the exception of cases of CML newly diagnosed during pregnancy, first-line treatment is a TKI. A short course of hydroxyurea may be given in symptomatic patients with high white blood cell or platelet counts while molecular and cytogenetic confirmation of the CML diagnosis is pending. Currently, four TKIs have been approved for first-line treatment by the FDA and EMA: imatinib (Glivec®, Novartis, or generic), dasatinib (Sprycel®, Bristol-Myers Squibb), nilotinib (Tasigna®, Novartis), and bosutinib (Bosulif®, Pfizer). These are available almost everywhere, though with some differences in indications, dosing, and reimbursement. A fifth TKI, radotinib (Supect®, Dae Wong Pharmaceuticals) has been approved in South Korea only. Dasatinib, nilotinib, bosutinib, and radotinib have been tested against imatinib in company-sponsored randomized trials. They have never been tested against each other. The results of these trials have provided the basis for approval of these TKIs in the first-line setting. Comparisons among these trials, and of these trials with academic studies, are difficult because of differences in protocols and methods of evaluation [33].

### Imatinib

Imatinib, the first generation TKI was tested in the IRIS study, where it showed higher rates of cytogenetic and molecular responses compared with a combination of recombinant interferon alpha (IFNα) and low-dose cytarabine [34] and better progression-free survival (PFS) and OS [35]. Imatinib immediately became the first-line choice for the treatment of CML. Three academic trials [36–39] rapidly confirmed and extended IRIS by testing imatinib in combination with IFNα or low-dose cytarabine and at higher dosage, providing benchmarks for deep molecular responses, and demonstrating normal life expectancy in most patients. Additional information on imatinib came from other academic prospective and retrospective studies as well as from population-based registries [1, 2]. Imatinib served as control arm in six prospective pharmaceutical company-sponsored trials testing dasatinib, nilotinib, bosutinib, and radotinib [40–45] and in three prospective academic trials testing dasatinib [46–48].

The standard dose of imatinib is 400 mg once daily. In CP a lower dose of 300 mg can be used if 400 mg is not tolerated and the response is optimal. A dose of 400 mg twice daily can be used in AP, but in patients with progression to more advanced disease a change to second generation TKI (2GTKI) is recommended.

Following imatinib treatment, early molecular response rates at 3 and 6 months (BCR-ABL1 ≤ 10% IS) range between 60 and 80%. At one and 5 years, MMR rates range between 20–59% and 60–80%, respectively. The 5-year probability of achieving a DMR (MR^4^ or deeper) ranges between 35 and 68%. Changes of imatinib, mostly to another TKI, range from 26.5% within 10 years [4] to as high as 37% and 50% within 5 years [40, 41]. Five-year PFS and OS range between 80–90% and 90–95%, respectively. Ten-year OS ranges between 82 and 85% with a leukemia-related death rate of about 6% [4, 35].

No absolute contraindications have been reported for the use of imatinib, and no life-threatening complications have surfaced although patients with a low cardiac ejection fraction and a low glomerular filtration rate should be observed carefully for related organ toxicity [4, 35]. The most frequent causes of poor tolerance are early fluid retention, gastrointestinal symptoms, muscle cramps, joint pain, skin rash, and fatigue [37, 49]. Many of these adverse events resolve over time or after a brief drug holiday. A dose reduction may be considered in patients who achieved an MMR [50].

### Dasatinib

Dasatinib is a 2GTKI, more potent than imatinib and active against several imatinib-resistant BCR-ABL1 mutants (Table 5). The DASISION trial [40], comparing dasatinib, 100 mg once daily, with imatinib, 400 mg once daily, with a minimum follow-up of 5 years, showed that with dasatinib the early molecular response rate (84%), the MMR rate by 1 year (46%), the 5-year cumulative probabilities of achieving MMR (76%) and MR^4.5^ (42%) were significantly higher, but PFS and OS were similar (86% vs. 85% and 91% vs. 95%, respectively). The rate of changing from either primary treatment was similar, 39% vs. 37%. Three academic studies with 1–5 years follow-up reported similar data [46–48].

**Table 5:** Recommended tyrosine kinase inhibitors in case of BCR-ABL1 resistance mutations.

T315I	Ponatinib
F317L/V/I/C, T315A	Nilotinib, bosutinib^a^, or ponatinib
V299L	Nilotinib or ponatinib
Y253H, E255V/K, F359V/I/C	Dasatinib, bosutinib^a^, or ponatinib

^a^There are limited data available regarding mutations associated with clinical resistance to bosutinib in vivo. Some in vitro data suggest that the E255K and, to a lesser extent, the E255V mutation, might be poorly sensitive to bosutinib.

The approved CP dose of dasatinib is 100 mg once daily for CP, and 70 mg twice daily for advanced phase CML. An increase of the dose is not recommended. Because of fewer side-effects at similar response rates in CP, a dose as low as 50 mg is an option [51].

Dasatinib has pleuro-pulmonary toxicity so that respiratory failure and previous or concomitant pleuro-pulmonary or pericardial diseases are strong contraindications to the use of dasatinib as first-line management. Chronic complications include recurrent pleural effusions in as many as 37% of patients; this may occur even after years of previously uncomplicated treatment. Rarely pulmonary arterial hypertension is observed [40]. Aside from these side-effects, tolerability was the same as with imatinib [40].

### Nilotinib

Nilotinib, another 2GTKI that is more potent than imatinib, inhibits several imatinib-resistant BCR-ABL1 mutants (Table 5). The ENESTnd trial [41] compared nilotinib, 300 mg twice daily with imatinib, 400 mg once daily, now with a minimum follow-up of 10 years [52]. Following nilotinib treatment, the 5- and 10-year cumulative probabilities for achieving MMR were 77% and 82.6%, MR^4^ (66% and 73%) and MR^4.5^ (54% and 64%), respectively. Although these results were significantly better than with imatinib, nevertheless 5- and 10-year OS were similar (94% vs. 92% and 87.6% vs. 88.3%, respectively). The rates of changing primary treatment were 40% for nilotinib and 50% for imatinib. In a phase II study of 73 newly diagnosed CP patients, the 10-year intention to treat outcome was 98% MMR, 76% MR^4^, and 94% OS [53]. Another randomized study of nilotinib versus imatinib in first-line, ENESTchina [42], revealed similar response data by 1 year; approximately the same results have been reported in other company sponsored (ENEST1st [54],) and academic studies [3, 55].

The approved dose of nilotinib is 300 mg twice daily in first-line treatment and 400 mg twice daily beyond first-line. A dose of more than 300 mg twice daily should be used cautiously owing to a dose-related increase in the risk of cardiovascular side-effects [41, 52, 53, 55].

Cardiovascular events occurred in about 20% of patients over a 10-year period [52] compared with 5% with imatinib. A history of either coronary heart disease, cerebrovascular accidents, or peripheral arterio-occlusive disease represents a strong contraindication for using nilotinib as first-line therapy. Patients with hypertension, hypercholesterolemia, and diabetes mellitus may also be at increased risk and, because pancreatitis has been reported in about 5% of treated patients, a history of pancreatitis also represents a contraindication to nilotinib use.

### Bosutinib

Bosutinib is the third 2GTKI that is more potent than imatinib. It also inhibits several BCR-ABL1 mutants (Table 5). The registration trial BFORE compared bosutinib with imatinib, each at 400 mg once daily [43]. After a median follow-up of <2 years, early molecular response rate (75%), and 1-year MMR rate (47%) with bosutinib were significantly higher than with imatinib [43].

The approved dose is 400 mg once daily in first-line, and 500 mg once daily [44] in second-line. An increase of these doses is not advised. A lower dose may be considered if 500 or 400 mg are not tolerated and the response remains optimal. No relevant comorbidities and no strong contraindications have been identified. Typically transient diarrhea occurs in as many as 30% of patients which can be an annoying side-effect. Transient elevations of transaminases may occur, mostly in the first weeks to months of treatment.

### Radotinib

Radotinib is a fourth 2GTKI that has been approved in first-line for South Korea, but this drug has not been reviewed by the FDA or the EMA. Radotinib is structurally very similar to nilotinib and exhibits an almost identical activity profile against BCR-ABL1 mutants. The registration trial (RERISE) [45] and the 4-year update [56] indicated that molecular response rates were significantly higher with radotinib at 300 mg twice daily than with imatinib. No relevant comorbidities or strong contraindications have been reported, thus far. A frequent increase of transaminase levels has been noted.

### Interferon α (IFNα)

In the pre-TKI era, IFNα was the treatment of choice. With the advent of pegylated (PEG) formulations that require less frequent administration and have improved efficacy and tolerability, IFNα may re-emerge as a therapeutic option in CP-CML [57]. Combinations of 2GTKIs with PEG-IFNα suggest some benefits compared with historical controls in small single arm studies [58, 59]. PEG-IFNα in combination with imatinib produced deeper and faster responses in two randomized trials, although no long-term survival benefit was demonstrated [38, 60]. Randomized investigational trials of PEG-IFNα in combination with nilotinib are ongoing. The hope in these studies is that PEG-IFNα may increase the proportion of patients qualifying for TFR because of its immunological effect.

### Generics

Generic imatinib [61–63] is now available worldwide and generic dasatinib is soon to be released. Quality generics have advantages in that the cost of therapy is often significantly reduced, making the drugs more affordable and hence more available to patients with limited resources. This should also help with compliance issues, because patients are delinquent in self-medication when required to pay in part or in full for their drugs [64, 65].

As long as a generic drug meets the national standards of the country involved in terms of quality of production, bioavailability and efficacy, then it is an acceptable alternative to the brand product. Generic and brand product dosing should be the same. Monitoring the response to generics must be the same as with branded drugs, but if a patient is changed from a brand to a generic product, then enhanced vigilance for the first 6 months in terms of sustaining response and observing for new adverse events is advisable. It is recommended that the patient continues to use the same generic brand if at all possible, in order to avoid potential side-effects due to changes in drug structure, bioavailability and excipient.

### TKI costs and cost effectiveness

Since adherence to life-long TKI therapy is critical for most patients with CML [64, 65], TKI costs and cost effectiveness have become important issues for patients and society, which is justifiably involved in drug costs. Life-long costs are an important variable when deciding on a first-line TKI and changing to alternative drugs in the course of treatment. The potential for a prolonged TFR and discontinuation of all treatment are also involved in these issues. Several studies have analyzed cost effectiveness of frontline treatments in various scenarios including strategies to increase the rate of sustained DMR to potentially increase the rate of TFR [66–69]. The conclusion is that generic imatinib is the cost-effective initial treatment strategy for CML-CP. Until 2GTKI lose patent protection, cost effectiveness will continue to be an important issue in choosing first-line TKI because 80% of patients will never achieve a TFR.

## Second-line treatment

Treatment is often changed from the first-line TKI for several reasons. In cases of failure/resistance, the change is mandatory and must be accompanied by investigation of BCR-ABL1 KD-mutations (Table 5) [70–73]. In case of intolerance and treatment related complications, the decision to change is in part subjective, depending upon the patient, physician, options for supportive care, and also upon the level of response. In case of warning, the change is optional, depending on the decision of pursuing a policy of early treatment discontinuation as well as on patient-related factors, such as age, lifestyle, comorbidities, and tolerance. In the absence of BCR-ABL1 KD-mutations, there can be no clear recommendation for any particular 2GTKI: all second-line TKIs are effective, but there are no studies comparing the TKIs with each other. The criteria for the choice of the second-line TKI are almost entirely patient-related and depend on age, comorbidities, toxicity of first TKI, etc. All patients need to continue daily TKIs in CP and in AP/BP. In the absence of alternatives, a TKI should be continued even among CP-patients who do not achieve cytogenetic response, because TKI still seem to confer a survival advantage in this situation although there are no systematic studies in this scenario or biologic studies to explain this observation. All approved first-line TKIs are available for second-line use in the doses reported previously.

The definition of the response (milestones) to second-line treatment should be the same as to first-line treatment (Table 4).

## Treatment beyond second-line

The definition of an acceptable response to third, fourth, or fifth-line treatment cannot be formalized, but a BCR-ABL1 transcript level >1% or a cytogenetic response less than complete (Ph+ >0%) are insufficient for optimal survival. There are no comparative studies and the choice of TKI should be guided by the sensitivity profile of specific BCR-ABL1 KD-mutations if possible, and, in particular T315I where only ponatinib is efficacious (Table 5). Suboptimal response to two or more TKIs should lead to prompt consideration of an allogeneic stem cell transplantation (allo-SCT).

### Ponatinib

Ponatinib (Iclusig®, Takeda/Incyte) is a third generation TKI more potent than all other TKIs [74–77]. Ponatinib has been approved for patients with the BCR-ABL1^T315I^ mutation and for patients with CML resistant to two or more TKIs [74]. In patients with resistance to a 2GTKI without specific mutations ponatinib is preferred rather than an alternative 2GTKI unless cardiovascular risk factors preclude its use [75, 77].

The FDA approved dose of ponatinib is 45 mg once daily. Cardiovascular toxicity can occur in about 30% of recipients [75] and may be dose-related; the panel advises starting at a lower dose (30 mg or 15 mg daily) for patients with lesser degrees of resistance or multiple intolerances, especially those with an increased cardiovascular risk profile. The dose is increased only if needed. The panel recommends starting with 45 mg once daily only in patients with T315I, or compound mutations, or progression to an advanced phase. Control of hypertension, hyperlipidemia and diabetes and smoking cessation should be emphasized to possibly reduce the risk of arterial occlusion events (AOE). The benefit of prophylactic acetyl salicylic acid or anticoagulation is uncertain. Preliminary data suggest that if a CCyR or MMR is achieved, the daily dose can be decreased to 15 mg daily followed by careful monitoring of disease and toxicity. Allo-SCT should be considered, if appropriate.

## Toxicity, side-effects, and complications

The toxicity of TKIs has never been the primary endpoint of clinical studies and was not patient reported. Rather, it was evaluated based on the reports of health professionals, using severity scales designed to evaluate the acute toxicity of cytotoxic cancer chemotherapy in a clinical setting different from the setting of chronic TKI therapy [78]. Therefore, it is still difficult to provide a comparative evaluation of the frequency and the severity of toxicities [49]. Toxicity has been always reported under the general heading of “adverse events”, but two important distinctions should be made. First, hematologic and non-hematologic “adverse events” should be considered separately. Hematologic adverse events (neutropenia, thrombocytopenia, anemia) are usually limited to the first treatment period, may require temporary dose adaptation, are rarely a cause of treatment changes, and very rarely a cause of complications. There are no data showing that the hematologic toxicity of the different TKIs is different, though it may be possible [49]. Second, non-hematologic “adverse events” should be divided into “side-effects” that affect tolerability and life quality and cause a treatment change in as many as 30% of patients, and in “complications” that require a treatment change in up to 15% of patients, because they may affect significantly patient health and life quality, and may sometimes cause death.

All TKIs have toxicities which may cause clinically relevant complications [49, 79]. These must be considered when selecting a TKI for a patient with comorbidities [80], because some are contraindications for using a specific TKI first-line. Previous or concomitant arteriovascular disease represents a strong contraindication to nilotinib first-line and ponatinib second or third line [41, 52, 55, 61, 62, 77, 79, 81, 82], unless there is unique need. Respiratory failure and previous or concomitant pleuro-pulmonary disease are strong contraindications to dasatinib first line. Imatinib should be withheld in patients with significant renal impairment. No other strong contraindications to imatinib or to bosutinib have been identified.

The excess risk of AOE is highest with ponatinib [74–77, 82], followed by nilotinib [79], and substantially lower with other TKIs. For any TKI, QTcF should be measured by electrocardiography prior to commencing and subsequently before using any other agent known to increase the QT interval [83, 84].

Pleural effusion is primarily associated with dasatinib, with a 5-year cumulative incidence of 37%. Risk factors include older age, twice daily dosing, previous or concomitant cardiac disease or autoimmune disorders, hypertension, hypercholesterolemia, and advanced phase CML [85].

Diarrhea or constipation may occur with any TKI. Diarrhea is particularly common with bosutinib, though this is usually self-limited and may be less problematic with the 400 mg dose once daily and with concurrent or pre-emptive treatment with loperamide.

Hepatotoxicity may occur with any TKI, but particularly with bosutinib and nilotinib, although typically this consists only of an increase of transaminases rather than more serious evidence of drug-induced liver injury. Nilotinib competes for UDP glucuronyl transferase and may result in an unconjugated hyperbilirubinemia in patients with Gilbert´s disease without clinical impact.

Hyperglycemia occurs mainly with nilotinib. Patients with controlled diabetes mellitus type II or pre-diabetes may receive nilotinib if strict glucose monitoring is followed. Nilotinib may also increase serum cholesterol levels associated with AOE.

Cytopenias often occur during the first weeks of treatment with any TKI reflecting the on-target antileukemic action. If there is slow or poor repopulation by normal hematopoietic cells, cytopenias are likely to occur with any TKI and, if severe, may preclude the delivery of an adequate TKI-dose. These patients are candidates for supportive care with growth factors and may even require temporary support with blood products. If the cytopenias cannot be resolved, the patient should be considered for allo-SCT. Dasatinib, a potent SRC-inhibitor, may affect platelet function leading to a bleeding tendency disproportionate to the platelet count.

Elevations of serum lipase, sometimes with clinical manifestations of pancreatitis, have been reported after treatment with nilotinib and bosutinib. Therefore, in patients with a history of pancreatitis other TKIs are preferred.

## Treatment options for resistant BCR-ABL1 mutations

Resistance to imatinib occurs in 10–15%, and to 2GTKI in <10% of patients in first-line treatment. In some patients, failure to respond may be related to poor or intermittent compliance with treatment, and these patients should be questioned closely about their adherence to the recommended dose and schedule of medication. Resistance may be caused by a BCR-ABL1 KD-mutation coding for a BCR-ABL1 protein that is poorly inhibited by TKIs. Mutations account for resistance in about one-third of resistant CP patients, and in about two-thirds of resistant AP and BP patients. Alternative mechanisms of resistance include clonal evolution (additional chromosomal aberrations, ACA) and the activation of BCR-ABL1 independent pathways. Not all ACA indicate progression equally. A cytogenetic risk classification has therefore been proposed to allow risk-based treatment adaptation [17, 86].

BCR-ABL1 mutations can be detected with sensitivities of about 20% by Sanger sequencing and of about 3% by NGS. The greater sensitivity of NGS enables the early detection of clinically relevant BCR-ABL1 resistance mutations [87, 88]. NGS is the recommended technology to detect BCR-ABL1 resistance mutations in patients not responding adequately to TKI.

In about two-thirds of resistant CP patients and in about one-third of resistant AP and BP patients, a mutation is neither detected nor is it the only cause of resistance, after compliance has been verified. In the future, analyzing the genome and expression profiles of resistant CML cells may lead to identifying somatic mutations [89–91] as early signs of progression and to a genomically based risk classification with the potential for non-BCR-ABL1 targeted therapy for resistant patients.

The phenomenon of the so-called operational cure, namely the persistence of detectable leukemic cells while either on-treatment or off-treatment in DMR, differs from resistance. This is likely governed by different factors, such as the immune control of minimal residual disease and the type of BCR-ABL1 transcript, because e13a2 (b2a2) cells are more persistent than e14a2 (b3a2) transcripts [92].

## Treatment of advanced-phase CML

Progression of CML to end-phase has fortunately become a rare event. End-phase CML comprises early progression with emerging high-risk ACA and late progression with failing hematopoiesis and blast cell proliferation. BP is a late feature of progression [93]. Currently, diagnosis rests on the percentage of blasts (20% or 30%) in blood or marrow [6, 94]. Not all patients dying of CML reach the BP-defining blast levels [95]. Once BP has occurred, survival is generally <1 year with death due to infection or bleeding.

Early indicators of progression are the appearance of ACA [16–18], somatic mutations [90, 91], and occasionally clinical deterioration without obvious explanation. Flow cytometry distinguishes between lymphoid and myeloid BP allowing appropriate selection of treatment. Lymphoid BP has more treatment options and a better outcome than myeloid BP.

After KD-mutation analysis, treatment consists of intensive combination chemotherapy with or without a TKI in preparation for a prompt allo-SCT if possible (Table 6). In patients with resistance to a 2GTKI without specific mutations ponatinib is preferred over change of 2GTKI, unless cardiovascular risk factors are present [77]. In patients who cannot tolerate intensive chemotherapy regimens, a more palliative approach with less intensive therapy according to immunophenotype should be considered.

**Table 6:** Management strategy recommendations for end-phase CML.

Prevention by elimination of BCR-ABL1	Assurance of effective TKI treatment
Early: emergence of high-risk ACA	Observe closely, consider intensification of treatment (ponatinib, early allo-SCT)
Primary blast phase	Start with imatinib, change to a 2GTKI according to KD-mutation profile.
Resistance to 2GTKI (first or second line)	Ponatinib or experimental agent. Assessment for allo-SCT, donor search.
Failure to ponatinib	High risk of progression, early allo-SCT recommended
Accelerated phase	To be treated as high-risk patients; proceed to allo-SCT if response not optimal.
Progress to blast phase	Attempt at return to CP2. Outcome with currently available TKI poor. Addition of chemotherapy based on AML regimens for myeloid BP (such as dasatinib or ponatinib + FLAG-IDA) or ALL regimens for lymphoid BP (such as imatinib or dasatinib + hyperfractionated CVAD) recommended. Choice of TKI should be based on prior therapy and BCR-ABL1 KD-mutational status. After CP2 is achieved proceed to allo-SCT without delay.

*ACA* additional chromosomal aberrations, *allo-SCT* allogeneic stem cell transplantation, *2GTKI* second generation tyrosine kinase inhibitor, *CP1* first chronic phase, *CP2* second chronic phase, *BP* blast phase, *AML* acute myeloblastic leukemia, *ALL* acute lymphoblastic leukemia.

## Allogeneic stem cell transplantation (allo-SCT)

In first CP (CP1) allo-SCT still has a place in managing the small number of patients with disease resistant or intolerant to multiple TKIs, and for the very rare patient with inadequate recovery of normal hematopoiesis. In resource-poor countries, allo-SCT may have priority over TKI treatment as the one-time expenses of transplantation are more economical than life-long drug costs.

For a patient who is resistant to the initial 2GTKI given either as first or second-line therapy, the chance of achieving a durable response to an alternative 2GTKI is low, and ponatinib or an experimental agent should be considered (Table 6). At this juncture the patient should also be assessed for allo-SCT and a donor search initiated. Failure to respond to ponatinib after 3 months' treatment indicates a patient at high risk of progression, and an early transplant is indicated. The patient should be assessed for allo-SCT and a donor search initiated.

A patient presenting in AP should be treated as a high-risk patient, becoming eligible for allo-SCT if the response is not optimal. A patient progressing to AP during treatment should immediately be considered for allo-SCT.

For patients presenting in, or progressing to BP, the long-term outcome with any of the currently available TKI is poor [96]; every effort should be made to offer allo-SCT after initial control of their disease. Patients returning to a second CP (CP2) before allo-SCT have improved transplantation outcomes [97, 98]. The addition of a TKI to chemotherapy-based AML [99, 100] or ALL regimens [101] improves the chance of achieving CP2. The choice of TKI should be based on prior therapies and BCR-ABL1 KD-mutational status. If CP2 is achieved, patients should proceed to allo-SCT without delay, as PFS in BP is low and time to allo-SCT plays a crucial role [102]. Transplantation in frank BP is not recommended.

## Quality of life

TKIs have improved patient outcomes to near-normal, and thereby survival, so that quality of life during treatment is clearly important. There are few quantitative studies, and many focus on the comparisons of tolerability of different TKIs which is only one aspect of life quality [103–109]. Since all patients receive TKIs for many years, and the majority currently remain on-treatment indefinitely, we encourage additional research in this area and the use of patient-reported outcome questionnaires which may provide guidance about quantifying and addressing chronic issues faced by CML patients [12].

## Treatment-free remission

A significant proportion of patients will achieve a DMR defined as BCR-ABL1 levels of MR^4^ and MR^4.5^ on the IS with the familiar TKIs. Table 7 lists the benchmarks of DMR that can be expected by 5 and 10 years [36, 37, 40, 41, 52]. Five-year follow-up of first-line bosutinib is not yet available. An attempt at treatment discontinuation can be considered, if sustained DMR of sufficiently long duration has been achieved.

**Table 7:** Cumulative incidence of deep molecular response (MR^4^ and MR^4.5^) with imatinib, nilotinib, and dasatinib by 5 and 10 years.

Study		5 years (%)	10 years (%)
CML-Study IV^a^, [36, 37]	Imatinib MR^4^	68	81
	Imatinib MR^4.5^	53	72
ENESTnd^b^, [41, 52]	Nilotinib MR^4^	66	73
	Nilotinib MR^4.5^	54	64
	Imatinib MR^4^	42	56
	Imatinib MR^4.5^	35	45
Dasision^c^, [40]	Dasatinib MR^4.5^	42	NA
	Imatinib MR^4.5^	33	NA

DMR rates of these trials cannot be directly compared owing to different methods of trial evaluation.

*NA* not available.

^a^Imatinib (*n* = 1442).

^b^Nilotinib 300 mg twice daily (*n* = 282), imatinib 400 mg daily (*n* = 283).

^c^Dasatinib 100 mg once daily (*n* = 259), imatinib 400 mg daily (*n* = 260).

The first prospective proof of concept for stopping TKI treatment was the Stop Imatinib 1 (STIM1) trial which showed that 38% of the patients maintained a molecular remission after a median follow-up of 77 months [110]. The eligibility criteria for stopping treatment was around MR^4.5^ sustained for the 2 years before stopping.

Since then, multiple trials have been conducted or are still ongoing, each of which used slightly different entry criteria and different triggers for restarting the TKI. A metaanalysis of 15 cohort studies including more than 500 patients after imatinib cessation demonstrated a mean molecular recurrence rate after imatinib cessation of 51% illustrating the high reproducibility of the results [111]. More recently, the large EURO-SKI study evaluated 755 patients with similar overall results [112]. A consistent inclusion criterion in most studies was a minimum duration of TKI therapy of 3 years and a sustained DMR of at least 1 year. Recent data suggest that the TFR success rate for patients with stable MMR, but not achieving DMR is significantly lower than for stable DMR [113]; this observation requires confirmation.

Of importance, more than 80% of recurrences occur within the first 6–8 months after stopping emphasizing the need for frequent monitoring and structured follow-up during this early period. Stopping treatment is a safe procedure at centers with access to high-quality molecular monitoring and with careful patient selection. Some patients otherwise eligible for TFR prefer to remain on therapy, and it is important that clinicians discuss the available data with patients before a TFR attempt is begun. Loss of MMR has been the trigger for restarting therapy in most studies [114]. Confirmation of loss of MMR on a second occasion is not considered necessary and could delay restarting therapy. It should be emphasized that some patients have fluctuating values between MMR and MR^4^ values which sometimes improve over time without restarting TKI; such patients require careful serial monitoring. About 90–95% of patients who experience molecular recurrence regain their initial molecular level after restarting TKI therapy. Usually, the same TKI is restarted, unless prior side-effects indicate a change. So far, very few of >3000 patients who have been followed in TFR trials have had unfavorable outcomes: one patient had loss of MMR due to mutation, one had loss of cytogenetic remission, and one had progression to BP, but it is likely that numbers increase with more patients observed over longer times.

Loss of MMR is uncommon after 1 year in TFR although continued long-term monitoring is recommended because the follow-up of all these studies is <10 years and it is unknown whether or how often late relapses might occur. The mechanism(s) which prevent recurrence are not understood and are under investigation focussing on possible immune-mediated control of residual disease.

After stopping nilotinib or dasatinib as either first or second-line therapy, the probability of maintaining TFR has been ~50% [115–117], similar to the results after stopping imatinib.

Different prognostic factors for TFR success have been reported. Longer durations of TKI therapy and DMR and prior treatment with IFNα were identified as important indicators for maintaining MMR at 6 months in EURO-SKI, the largest TFR trial so far; of these, duration of DMR seemed to be the most important factor [91].

A characteristic polymyalgia-like syndrome of musculoskeletal and/or joint pain beginning the first weeks or months after TKI discontinuation has been reported in about 20–30% of patients [118]. This phenomenon is likely due to undefined off-target effect(s) of the TKI. In most patients the symptoms are mild and self-limited, but some patients may require temporary treatment with acetaminophen, nonsteroidal anti-inflammatory drugs or in some instances a short course of oral corticosteroids.

The ELN panel recommendations for TKI discontinuation are summarized in Table 8 [119, 120].

**Table 8:** Requirements for tyrosine kinase inhibitor discontinuation.

Mandatory:
• CML in first CP only (data are lacking outside this setting)
• Motivated patient with structured communication
• Access to high quality quantitative PCR using the International Scale (IS) with rapid turn-around of PCR test results
• Patient’s agreement to more frequent monitoring after stopping treatment. This means monthly for the first 6 months, every 2 months for months 6–12, and every 3 months thereafter.
Minimal (stop allowed):
• First-line therapy or second-line if intolerance was the only reason for changing TKI
• Typical e13a2 or e14a2 BCR–ABL1 transcripts
• Duration of TKI therapy >5 years (>4 years for 2GTKI)
• Duration of DMR (MR^4^ or better) >2 years
• No prior treatment failure
Optimal (stop recommended for consideration):
• Duration of TKI therapy >5 years
• Duration of DMR > 3 years if MR^4^
• Duration of DMR > 2 years if MR^4.5^

The panel agrees that TFR is a new significant goal of CML management. It recommends consideration of TFR in appropriate patients after careful discussion employing the concept of shared decision making [121]. Treatment may be changed to 2GTKI to improve the depth of response in selected patients in whom DMR has not been reached. In special situations such as the motivated patient with a high priority for TFR, younger patients with low or intermediate-risk disease or women who wish to become pregnant, a change to 2GTKI is recommended for consideration.

## Pregnancy and parenting

Many patients of child-bearing potential seek advice regarding the effects of CML and TKI regarding fertility, pregnancy, and effects on offspring. Sensitive, informed communication is mandatory, as is good collaboration with obstetric colleagues.

For men taking imatinib, bosutinib, dasatinib, or nilotinib, there is no increased risk of congenital abnormalities in their offspring [122–127]. Data are sparse/absent for ponatinib and asciminib, respectively. Changes in sperm quality and morphology can be present at diagnosis and are unchanged after imatinib [128]. Therefore, men planning fatherhood do not need to discontinue treatment with imatinib or 2GTKI.

For women, management of CML occurring during pregnancy must be individualized. TKI treatment should be discontinued in the first trimester, as soon as pregnancy is confirmed. Fetal ultrasonography should be performed immediately. Options of continuing or discontinuing treatment and continuation of pregnancy or not should be exhaustively discussed.

The teratogenicity of TKI is due to off-target, most likely PDGFR, inhibition during organogenesis. The occurrence of hydrops fetalis when dasatinib was commenced in the second trimester [123] suggest all TKIs are contraindicated throughout pregnancy. Although imatinib has been used safely in the second and third trimesters, insufficient experience does not allow its routine use [126, 129].

Termination of the pregnancy is a consideration in more advanced disease. If the white blood cell count is low, CML treatment may not be required before delivery. Acetyl salicylic acid and/or low molecular weight heparin are indicated for thrombocytosis. Leucapheresis and/or IFNα are safe throughout gestation [122, 130]. Close collaboration with obstetricians and regular fetal ultrasound examinations are recommended. Low-level secretion of TKI in breast milk contraindicates their use during breast-feeding [131].

Women eligible for a trial of TFR can also safely discontinue their TKI in order to conceive. Management thereafter depends on maintenance or loss of MMR. Women who lose MMR and are pregnant are likely to reach term without a clinical need for restarting treatment. Women who lose MMR and are not yet pregnant should restart treatment, perhaps with a more potent TKI, and could try to discontinue again when deep molecular response has been re-established and maintained for the appropriate time. More challenging is the woman desiring pregnancy without a sustained DMR (often associated with older age and/or societal pressures). Possible solutions include substitution of TKI with IFNα or referral for alternative methods for conception.

## Discussion

New developments and findings prompted this fourth version of the ELN management recommendations. All randomized studies comparing imatinib 400 mg once daily with 2GTKIs, imatinib 400 mg with dose increase, or imatinib combined with IFNα or low-dose cytarabine have failed to improve OS. Although deeper molecular responses occurred more rapidly with 2GTKIs, this did not translate into better OS than with imatinib at a standard dose of 400 mg daily. These studies, however, provided greater insights in the safety and efficacy of the drugs, as well as benchmarks for molecular response as a basis for individualized treatment and eventually treatment discontinuation. The studies showed that survival has moved close to that of the general population. Now more patients die of CML-unrelated causes than from CML. Although no new serious late side-effects of imatinib have surfaced over 20 years, chronic low-grade fatigue and muscle cramps remain issues for many patients remaining on-treatment.

Another very important development has been the recognition that treatment can be successfully stopped in some patients if the duration of both treatment and DMR is sufficient to make TFR feasible for potential cure. The panel agreed that TFR is an important new goal of CML management which should be discussed with appropriate patients [121].

There was considerable discussion, but with no final consensus, regarding the advisability of changing therapy (usually from imatinib to a 2GTKI) in a patient with stable CCyR or MMR, but in whom the level of DMR (<MR^4^) was insufficient to warrant consideration of discontinuation. There is no information about the rate of successful TFR from large randomized trials with different initial treatment regimens addressing this specific issue.

The ENESTcmr trial [132, 133] provides some relevant information, however. In what was termed “persistent molecular disease”, those patients who had achieved CCyR were randomized to continuing imatinib or switching to nilotinib 400 mg twice daily. The rate of achieving MR^4.5^ was higher in the nilotinib recipients (54% vs. 45% [32% in imatinib patients who did not cross over to nilotinib]). But, only 57% of patients completed 48 months of nilotinib therapy and 13% of patients randomized to nilotinib experienced significant cardiovascular events. There was no information whether patients who reached the MR^4.5^ endpoint successfully achieved TFR subsequently. An ongoing randomized study, SUSTRENIM (NCT02602314), will provide information on TFR rates after patients were treated for 4 years with either nilotinib or imatinib (with change to nilotinib if treatment milestones are not reached). Treatment cessation will be offered after at least 1 year in MR^4^.

Advocates for a change of therapy argued that this approach could increase the number of patients in whom treatment can be stopped successfully. Arguments against pointed to the potentially more serious side-effects of 2GTKI, their increased costs and absent information about the number of patients who might actually benefit. Many more patients would be exposed to these side-effects compared with those expected to benefit and successfully reach TFR. In view of the limited data and the investigational requirements to answer these questions, the panel does not recommend a first-line 2GTKI, or a change to a 2GTKI, for faster DMR, but agrees that a change may be considered in selected patients. These might include: younger patients with low- or intermediate-risk disease, patients for whom TFR is a high priority, and women who wish to become pregnant. A change from 2GTKI to imatinib can be considered when no DMR is achieved within 5 years to avoid the risk of serious cumulative toxicity of 2GTKI.

Other important points:

1. The new ELTS risk score has been helpful in predicting the rate of death from CML in TKI-treated patients. The panel recommends using this score instead of the older ones. It is noteworthy that the ELTS score uses the same variables as the Sokal score, but with different weights.
2. Good quality molecular testing is now available worldwide replacing cytogenetic monitoring in most situations and obviating the need for bone marrow aspirations. If a change in therapy is under consideration because of inadequate response or disease progression (drug resistance), a bone marrow aspiration is recommended to assess for cytogenetic clonal evolution. BCR-ABL1 KD-mutation analysis should also be done in such circumstances.
3. New developments have also occurred in first-line therapy. Generic imatinib is now available worldwide at low cost. Dasatinib will soon become generic. Bosutinib is available first-line with a safety profile different from other 2GTKIs, and radotinib is approved first-line in Korea. Ponatinib remains the only TKI with activity against the T315I mutant, though it should not be used first-line. Trials are underway to assess whether the high level of response can be maintained with decreased cardiovascular risk using lower doses of the drug. Asciminib appears to be a drug of promise but more information is required [134].

Assuming continued compliance with medication, sustained CCyR (equivalent to ≤1% BCR-ABL1 on the IS), MMR, and DMR predict excellent long-term outcome. Progression to advanced phase is rare (<2 per thousand patient-years with MMR). Continued molecular monitoring is nevertheless recommended indefinitely.

1. Allo-SCT remains an important option for patients resistant or intolerant to two or more TKIs and for the ~6% of patients who still progress to BP.
2. All TKI are teratogenic and are contraindicated during pregnancy.
3. PEG-IFNα in combination with a TKI may also accelerate or deepen a molecular response, but currently remains investigational. Randomized comparisons of nilotinib with and without PEG-IFNα are underway.

The vision for the next 5 years is that more CML patients will successfully achieve TFR and that it will be possible to talk more confidently about the curability of CML.
